# An Exploration of Nurse Managers’ Understanding and Experiences of Using Evidence‐Informed Management in a Healthcare Facility in the United Arab Emirates: A Qualitative Study

**DOI:** 10.1155/jonm/8961395

**Published:** 2026-02-03

**Authors:** Diane Roobasoundhrie Chetty, Wilma Ten Ham-Baloyi, Dalena R. M. van Rooyen, Allison Herlene Du Plessis

**Affiliations:** ^1^ Department of Nursing Science, Faculty of Health Sciences, Nelson Mandela University, Gqeberha, South Africa, mandela.ac.za; ^2^ Department of Health Sciences, Faculty of Health Sciences, Nelson Mandela University, Gqeberha, South Africa, mandela.ac.za

**Keywords:** evidence-informed management, evidence utilisation, nurse managers

## Abstract

**Background:**

Despite the demonstrated benefits of evidence‐informed management, such as enhanced patient outcomes, improved working environments and reduced staff turnover, it is reported that nurse managers often fail to incorporate evidence into their management practices. This study explored and described the understanding and experiences of nurse managers regarding the utilisation of evidence‐informed management. Through an examination of their perspectives, the study aimed to identify the barriers and enablers in adopting evidence‐based practices in nursing management.

**Method:**

A qualitative explorative–descriptive–contextual study was conducted using five focus group discussions with twenty (*n* = 20) purposively selected nurse managers. Thematic analysis was used to analyse the data.

**Results:**

Three main themes emerged from the study, namely: (1) Nurse managers communicated the resources and requirements to support using evidence‐informed management practices; (2) nurse managers verbalised the need to establish a process or pathway to use evidence‐informed management; and (3) evidence‐informed management was found to improve organisational performance significantly.

**Conclusion:**

To facilitate the uptake of evidence by nurse managers to support their management practices, it is imperative to establish structures, processes and pathways that enable the utilisation of evidence. Additionally, this utilisation of evidence has been shown to improve the quality of patient outcomes and enhance the overall performance of the organisation. Implications for nursing management practice and education include continuous education and training to increase nurse managers’ knowledge and skills in utilising evidence. Moreover, nurse managers should adopt evidence utilisation as a daily management practice. Incorporating evidence‐based informed management into postgraduate education curriculum serves to better prepare prospective nurse managers.

## 1. Introduction

The increasing pressure of maintaining a balance between rapid technological advances, cost efficiency, effectiveness of service quality, patient‐centricity and sustainability continues to weigh heavily on healthcare organisations [1]. Nurse managers (NMs), in their role position, are strategically positioned in healthcare organisations and, with the utilisation of evidence‐informed management (EIMgt) practices, have the ability to positively influence organisations to achieve these goals [1]. EIMgt is characterised by the systematic application of the most reliable, relevant and recent collective evidence, focusing on conscientious seeking, appraising and applying evidence [2–4] to support management practices. This approach combines research‐based and non–research‐based evidence sources, including professional experience, customer and stakeholder feedback and organisational information [4]. Summarised, EIMgt advocates for the thoughtful consideration of collective evidence to effectively apply relevant and best evidence when addressing a specific event or situation.

NMs, as middle managers, hold a crucial position in aligning healthcare facility priorities with overarching organisational goals. They ensure high‐quality, safe, value‐driven services while optimising costs and resources [1, 5]. To do this effectively, NMs need to make agile, evidence‐informed decisions [6]. However, NMs often encounter challenges in utilising evidence for management practices [7].

While NMs advocate for evidence‐based clinical practice, they inconsistently apply evidence to management decisions, often proceeding from problem to solution without a structured process [5, 7]. The uniqueness of each situation, NM experience and contextual factors influence their evidence uptake [5, 7]. Recognising the complexities and subjectivity involved in NMs’ decision‐making is vital. Therefore, it is important to prioritise the process of using evidence over solely focusing on the outcomes.

Multiple factors hinder the uptake of evidence in management practice, stemming from the evidence itself, the practitioner and the research–practice gap. Practitioners face individual and organisational challenges [8]. A significant barrier is the lack of clarity and understanding of the practical application of EIMgt [3, 8, 9].

Evidence utilisation involves various approaches, such as seeking and critically appraising information from diverse sources, applying the evidence and evaluating outcomes [4]. While all organisations use evidence to some extent, managers may not realise they are practising EIMgt. The central concern revolves around whether they utilise a variety of systematically gathered, critically analysed and applied evidence. Characteristics of EIMgt‐oriented organisations include organised data collection, information management systems, access to academic literature, a culture of learning from errors and transparent communication [9].

Barriers include insufficient competencies in searching and appraising evidence, a lack of knowledge of evidence sources, cognitive biases, time constraints, limited resources and inadequate understanding of EIMgt [8]. NMs often lack high‐quality information and skills to apply scientific evidence [8–10]. Training is essential to improve these skills and promote EIMgt utilisation [8–10].

Individual management styles and lack of preparation pose challenges to effective EIMgt. Managers can be categorised into different types. These types include fact‐based, plan‐based, expertise‐based, ethics‐oriented and shareholder‐oriented managers. Effective management practices require a combination of these styles to adapt and navigate dynamic healthcare environments successfully [9, 10]. Personal characteristics, such as cognitive bias, further impede EIMgt. Diverse academic backgrounds influence NMs’ readiness to practise EIMgt [9].

Organisational structures, processes and culture significantly impact EIMgt utilisation. Leadership plays a crucial role in advocating for EIMgt by promoting evidence use, providing resources and modelling EIMgt behaviours [1, 3, 9]. The lack of such support can hinder NMs from adopting EIMgt practices.

The proliferation of knowledge has not matched its application in practice, creating a research–practice gap [9, 11]. To bridge this gap, there needs to be a balance between knowledge creation, dissemination and practical application [11]. The evidence pyramid, which ranks evidence levels, may not fully capture the diversity of useful information [9]. Integrating all types of evidence, including grey literature, is essential for effective EIMgt [9, 10].

Close collaboration between researchers and practitioners can support evidence uptake [9, 11]. Researchers should ensure that evidence is relevant, trustworthy and practical for practitioners [7, 9]. In the study context, NMs oversee multiple clinical units, using an electronic medical record system and business intelligence reports for support. However, in 2021, only three out of 199 NMs accessed an available organisational data dashboard, raising questions about their use of available data sources. Despite their access to resources, NMs often resort to manual data collection for decision‐making, leading to inconsistencies and a fragmented data perspective [8, 12]. This gap suggests a need for better integration of EIMgt practices among NMs. While there are some studies conducted within the healthcare context and the use of evidence in management practices, they focus on senior executive‐level healthcare managers [7, 10, 13]. There is a dearth of literature on utilising EIMgt by NMs at a middle management level and their understanding and experiences related to its use. As a result, the study aims to explore and describe NMs’ understanding and experiences in utilising EIMgt.

## 2. Methods

### 2.1. Study Design

To achieve the aim of the study, a qualitative research approach employing an exploratory–descriptive–contextual design was used. This study was part of a larger research study focusing on developing and evaluating a framework for NMs to facilitate the utilisation of EIMgt in a healthcare facility in the United Arab Emirates (UAE).

### 2.2. Setting

The study was conducted in a large tertiary healthcare facility in the UAE. The UAE has a diverse nursing workforce, with expatriate nurses comprising 98.5% and Emirati nurses making up only 1.2% as of 2018 [12]. The UAE government has set a target to raise the percentage of Emirati nurses and midwives to 4% by 2026, despite the current workforce having varying levels of clinical and academic preparation [14]. In the selected healthcare facility, middle managers are positioned between executives and frontline staff, playing a vital role in realising the organisation’s mission, vision, goals and objectives. Moreover, the NMs are responsible and accountable for supervising and achieving the functional and operational management goals of their clinical and work units. Their role includes planning, coordination and optimisation of resource utilisation in the delivery of patient care.

### 2.3. Participants and Recruitment

The research participants were NMs from a UAE healthcare facility who assumed supervisory roles within patient care units. As middle managers, NMs are responsible for planning, organising, managing and controlling the delivery of safe, high‐quality patient care [15, 16]. This positions them to benefit from EIMgt, making them the primary focus of the study, as opposed to senior executive‐level managers. The total population was twenty‐four (*n* = 24). Nonprobability, purposive sampling was adopted to draw participants from a population of NMs. The inclusion criteria for the sample were as follows: Eligible participants were required to be registered nurses (RNs) or registered midwives (RMs) serving as NMs or senior nurse leaders responsible for the functional and operational management of one or more patient care units or nursing work groups. Participants could be in permanent or acting positions, must have had at least six months of experience in a management or leadership role and supervised three or more staff members. The target sample was a maximum of twenty‐four (*n* = 24) and a minimum of ten (*n* = 10) participants and was determined by data saturation. The principle of data saturation, which involves the repetition of themes, no new information being shared and the emergence of no new codes from the data, was enforced [17].

Following approval of the study, eligible participants were contacted using a gatekeeper and invited to participate in the study via electronic mail [18]. The electronic correspondence contained information on the study, including potential risks, benefits, participant expectations and the informed consent form.

### 2.4. Data Collection

Five focus group discussion (FGD) sessions were conducted between March and June 2022 and included a total of twenty NM participants. Using FGDs to understand NMs’ use of EIMgt aligned well with the qualitative research design. It provides a contextual and diverse interpretation of each NM’s experience, capturing authentic voices, efficiently collecting data from many discussants simultaneously [19]. Moreover, the first author fostered a relaxed atmosphere, promoting engagement and dialogue [20], thereby yielding richer information that reflects the relationship between the participants’ experiences and their sociocultural context [21] in a hospital’s complex environment.

A facilitators’ guide with questions to stimulate exploration and discussion was created for the study based on the literature to facilitate the FGD. The main research question, ‘What validated framework can be developed and evaluated to facilitate utilisation of EIMgt by NMs in a healthcare facility in the UAE?’, was adopted to develop the FGD facilitators’ guide. The four authors reviewed and finalised the facilitators’ guide. The FGD facilitators’ guide included the following questions: *What informs or guides your management practice? What is your understanding of EIMgt? Share your experiences in using EIMgt; What facilitates the use of EIMgt? What hinders the use of EIMgt? If you had to design a framework to facilitate using EIMgt, what would you include?*

To prevent potential bias and researcher coercion stemming from the first author’s supervisory role, one facilitator and two fieldworkers were employed to assist with the facilitation of the FGDs. The facilitator, an RN working as a clinical resource nurse with a master’s degree, was responsible for leading and facilitating the FGD. The fieldworkers, both RNs working as nursing quality coordinators, one holding a Master’s degree while the other was pursuing a Master’s degree, coordinated the logistics of the FGD and scheduled FGDs with participants. Additionally, one of the fieldworkers took field notes to supplement audio recordings, capturing nonverbal cues for richer data descriptions [21]. The FGD venue was arranged within the study facility, as requested by the participants, and in an area with few interruptions and foot traffic. The first author oriented the fieldworkers to the study and role expectations through individual and collective meetings. Mock FGDs were conducted with the FGD facilitator and the fieldworkers to rehearse facilitation and practice interview techniques, such as probing, paraphrasing and summarising. These sessions also allowed the fieldworkers to test audio equipment, review logistics and build confidence in their roles. A pilot FGD was scheduled with three (*n* = 3) NMs. The data collected during the pilot FGD were analysed and reviewed by all four authors and included in the main study since no changes were required to the questions. The FGDs had a minimum of three (*n* = 3) and were limited to a maximum of six (*n* = 6) NMs per group. The FGDs were audio recorded, and all recordings were emailed to the first author within 12 h of the FGD. The FGDs were approximately 90 min in duration. Data saturation was reached by the fifth FGD, negating the need for further FGDs.

### 2.5. Data Analysis

The audio recordings were transcribed verbatim using the Otter.ai platform made available by the university. The data were analysed using the content analysis steps [22, 23]. The first author engaged in a comprehensive review of the FGDs, involving complete immersion in the recordings and transcripts to gain a thorough understanding of the content. The first author and an independent coder concurrently and independently analysed and coded the data to derive themes and reached a consensus on the themes. Subsequently, the themes were finalised after the review and discussion among all four authors. The findings of the FGDs were categorised into three main themes and subthemes and conceptualised within Donabedians’ structure–process–outcomes (SPO) model, which is commonly used to improve healthcare quality [24, 25].

**Figure 1:**
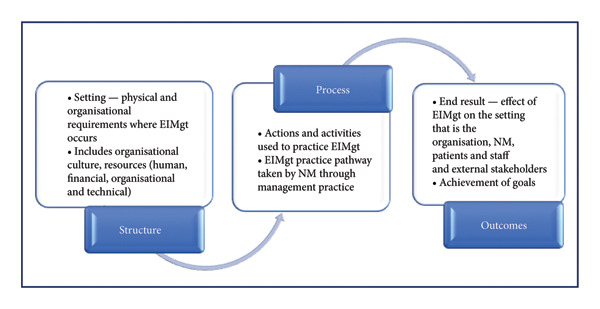
Donabedians’ structure–process–outcome model conceptualised to EIMgt (adapted after [24]).

Applied to the practice of EIMgt, the SPO model (Figure 1) demonstrates that robust, conducive and enabling organisational structures will facilitate enabling management actions, practices and pathways that will potentially lead to increasing the use of EIMgt by NMs. In addition to the main themes being aligned under the SPO concepts, the subthemes were categorised as Theme 1 aligned to the structure, while the subthemes can be categorised under *People, Position, Preparation and Power*. Theme 2 is aligned under processes, and the subthemes are categorised under *Pathway*. Theme 3 is aligned under outcomes, and the subthemes are grouped under *Performance*. Figure 1 illustrates this conceptual model applied to EIMgt.

### 2.6. Ethical Considerations

Following a full review of the study, ethical approval was received from the university and the study institutions’ Human Research Ethics Committee (H21‐HEA‐NUR‐023 and MF2058‐2022‐824, respectively). The research adhered to the ethical principles and guidelines outlined in the Belmont Report, which addresses the protection of human subjects in research [26]. Participants signed an informed consent. Confidentiality and anonymity of participants were ensured by using numerical codes and pseudonyms to deidentify participants. Electronic data were password‐protected, with access restricted to the first author. The first author and facilitators completed a Good Clinical Practice (GCP) certification. Privacy measures included organising the FGD venue in a low‐traffic area with minimal interruption and displaying a ‘do not disturb’ notice on the door during FGDs.

### 2.7. Trustworthiness

Research quality and trustworthiness were ensured by instituting utmost diligence and being meticulous in all procedures and processes employed in the course of conducting this study. Lincoln and Guba’s model of trustworthiness was applied, encompassing *credibility, confirmability, dependability, transferability and authenticity* [27], and other best practice strategies were implemented to ensure research quality and trustworthiness of the research [27]. Additionally, the inclusion of a pilot FGD, involving an independent coder during the data analysis and theme derivation process and holding discussions with other authors prior to reporting the findings contributed to the quality of the study. Credibility was ensured through transcribing the audio recordings using electronic software, thereby reducing transcription errors. Additionally, a coder was employed to identify themes from the FGD independently. Bracketing was used to reduce bias and preconception of the topic and data. Collaboration and robust discussions involving all four authors served to enhance the credibility of the study. Raw data, recordings, transcripts and data summaries of the FGD are available, presenting a transparent process.

## 3. Results

### 3.1. Participants’ Profile

Twenty (*n* = 20) NMs who met the established inclusion criteria for eligibility voluntarily consented to participate in the FGDs. The highest distribution of NMs (80%, *n* = 16) had less than 10 years of experience as an NM, while 20 per cent (*n* = 4) of the NMs had between 11 and 20 years of NM experience. Further, the majority of the NMs (45%, *n* = 9) held a degree at master’s level, while thirty per cent (*n* = 6) held a bachelor’s degree. Fifteen per cent (*n* = 3) held an advanced diploma, and ten per cent (*n* = 2) held a doctoral level qualification. All NMs met the minimum qualification and experience required for licensure as RNs or RMs, as mandated by the regulatory body of the hospital.

### 3.2. FGD Themes

The three main themes that emanated from the study are as follows: (1) NMs communicated resources and requirements to support using EIMgt practices; (2) NMs verbalised the need to establish a process or pathway to use EIMgt; and (3) EIMgt improves organisational performance (OP). Table 1 summarises the FGD themes and subthemes.

**Table 1:** FGD themes and subthemes.

Theme	Subtheme
Structure 1. NMs communicated resources and requirements to support using EIMgt practices.	People, Position, Preparation and Power 1.1 Experience, academic qualifications and continuous professional development. 1.2 Access to a variety of resources, instruments and tools. 1.3 Empowerment to implement evidence‐informed initiatives. 1.4 Engagement from senior leadership and bedside nursing staff.

Process 2. NMs verbalised the need to establish a process or pathway to use EIMgt.	Pathway 2.1 Some NMs have limited comprehension of EIMgt. 2.2 Some NMs feel overwhelmed by the large quantity of evidence and need to prioritise it. 2.3 NMs require training, competencies and skills in using EIMgt. 2.4 NMs shared ideas on how to develop a framework to use EIMgt.

Outcome 3. EIMgt improves OP.	Performance 3.1 Patient outcomes are positively influenced when NMs use EIMgt. 3.2 The outcomes of NM performance by using EIMgt must be measured. 3.3 Nursing staff outcomes are influenced when NMs use EIMgt.

The three main themes and subthemes are presented in Table 1.

**Figure 2:**
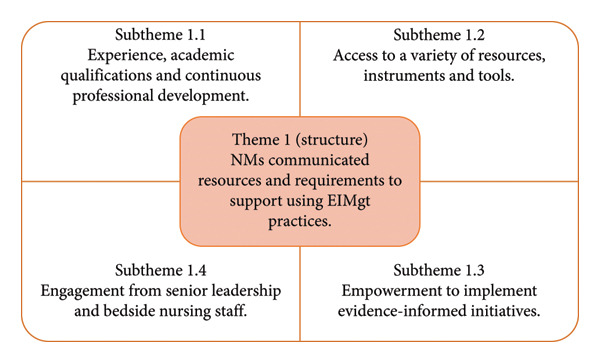
Summary of FGD Theme 1 and the subthemes.

Each of the three main themes and subthemes will be presented. Figure 2 displays the summary of FGD Theme 1 and the related subthemes.

#### 3.2.1. Theme 1: NMs Communicated Resources That Are Needed to Support Them in Using Evidence to Inform Their Management Practice

There was agreement from the NMs that the organisation has resources available to support them in using EIMgt. These resources include materials, financial aid, personnel and their capabilities and facilities [28, 29]. Together, these resources form an integral connection, influencing NMs’ ability to use evidence positively and negatively.

##### 3.2.1.1. Subtheme 1.1: NMs Are Experienced, Academically Qualified and Continued Their Professional Development to Support Their Role

All NMs are licensed as RNs or RMs, meeting the professional qualification requirements of the regulatory body, in addition to the organisation’s job description for an NM position. NMs described attending continuing professional development courses to prepare for and receive ongoing support from the organisation for their role and responsibilities as an NM.

‘*…I’ve been doing this management of the … for the past 20 years…’*

*‘…if you were involved with the same situation many times …you go back to see how you dealt with it…you learn from that experience…′*

*‘… I also had the opportunity to complete some postgraduate short courses from different platforms …’*

##### 3.2.1.2. Subtheme 1.2: NMs Have Access to a Variety of Resources, Instruments and Tools to Support Using Evidence to Inform Their Management Practice

NMs listed and provided a thorough explanation of various information sources, in the form of products and platforms, available to support EIMgt. Organisational information and experience were predominantly cited by NMs as the go‐to source of information. These sources included clinical performance indicators, patient satisfaction results and operational data, such as length of stay, case‐mix index, nursing productivity and staff turnover. Other sources of information that informed their practice included data from patients and staff. This related information resulted from patient satisfaction results, complaints and compliments and staff satisfaction feedback.

*‘I have to check data, statistics of what is happening in my unit. I need to know what is the gap… could take those from different dashboards from different results of KPI [Key performance indicators] from then I could plan my strategies.’*

*‘We have customer satisfaction data…Press Ganey’ [A company that offers an online platform for healthcare organisations to measure and improve patient and staff satisfaction]*

*‘There’s a lot of support at this hospital. And I don’t think you can ever be stuck, there’s always somebody [a professional] you can go to’.*

*‘… we can get it from colleagues who are more experienced or have the experience more than you it doesn’t matter the years of experience. Maybe they exposed to that kind of issue more than you…’*

*‘… because I didn’t have any experience … what helped me was the 70 20 10 model, which is the 70% of what you learn in your own experience. 20% is with interactions with people, and 10% is through, you know, studying and knowledge…’*

*‘… when you translate it to the practical side, sometimes we have challenges because theory sometimes different than in practice… you have to ensure that you have to do some customisation, which can fit your team, your units, how the people will accept it…’*

*‘…what guides me actually is my evidence-based practice, I do have to go back to the research to go back to the latest literature and review and take it from there …’*

##### 3.2.1.3. Subtheme 1.3: NM Empowerment Is Important for Implementing Initiatives Informed by Evidence

The FGDs garnered a mixed sense of NM empowerment. Although some NMs voiced positive responses regarding their ability to implement initiatives to support changes in operational processes and organisational indicators, other NMs expressed mixed feelings, some of whom expressed frustrations in not being able to execute this.

*‘…it was unfortunately proven that we have a lot of excess staff based on the methodology of calculation…so I went out and did a lot of googling… let’s say evidence, I developed a kind of a new methodology of calculation… it is really adopted … ’*

*‘…they implemented a new zero-based budget strategy. Unfortunately, maybe the new zero-based budget doesn’t reflect our actual need in our real situation. So as my colleague said, we need to go with the flow. …’*

*‘…the main challenges coming from senior management, … of course, they’re looking at the bigger picture, they look at finance, the budget and everything. So even though you have the evidence, it’s not always that you get things…’*

##### 3.2.1.4. Subtheme 1.4: Engagement From Senior Leadership and Nursing Staff Providing Bedside Care Is Critical If the Use of EIMgt Is to Be Successful

NMs perceived a deficit in engagement behaviours from senior leadership, such as the lack of showing support and resistance to implementing evidence‐informed initiatives. These were specifically noted as challenges by the NMs.

*‘…but sometimes when you learn something new, you’ve just got to make sure that the organisation is also behind it as well…. which actually seems great in theory, but seems to contradict the style, or the leadership of the management of the organisation, that might cause challenges…’*

*‘…in my opinion, the most important facilitator is to have top management who belief in evidence based …management believe and adopt it as a vison and strategy…top management words are very powerful… Right?’*

*‘…I think one of the challenges … it’s really the buy in, and also the lack of support…. you have to introduce change; you will sometimes receive resistance from the staff…you have to create an awareness… the change and how can it benefit them and their patients…”*

#### 3.2.2. Theme 2: NMs Verbalised the Need to Establish a Process or Pathway to Use EIMgt

**Figure 3:**
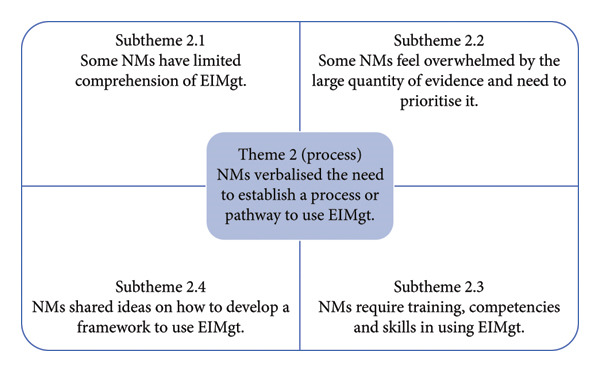
Summary of FGD Theme 2 and the subthemes.

In describing their experiences in using EIMgt, there was no clear discernible pathway or approach that NMs consistently and systematically used in their daily management practices. The FGDs revealed that NMs exhibited varied management styles, responses and reactions when dealing with issues and opportunities for improvement. Figure 3 is a summary of FGD Theme 2 and the related subthemes.

*‘…as management we don’t have [a] specific design…every manager they have their rules and regulation and style to solve it…’*

*‘…70% of my learning is from experience…so I use that a lot… …some managers are micromanager…some are democratic… how you’re dealing with everyday situations and also your own personality…’*

*‘…we’ve got experience…may have policies and procedures…you have to be quite adaptive and how you practice your management…it’s your management style as well…they go hand in hand…’*

##### 3.2.2.1. Subtheme 2.1: NMs Use Evidence to Support Their Management Practice; However, Some NMs Have Limited Comprehension About EIMgt

NMs were initially hesitant in responding about their understanding of EIMgt and explained components of EIMgt from a terminology perspective.

*‘… evidence, informed things that are being shared by the organisation, and then being informed to you to guide your, your practice with regards to managing your unit, …, whatever is in the organisation, the systems that are currently being practised…’*

*‘…this is managing with evidence through Theory and Practice, applying theoretical practices into applying theory into practice. And we learned that throughout the whole of our nursing from the very beginning, so I think it continues on into management…’*

*‘…this is part of the making decision to conscientious, explicit, judicious and use the result, very strong, you know, evidence available, your know you’ll have by your sources by asking gathering information…’*

*‘…to my understanding in some kind of scientific approach in towards…a combination relation between managerial I would say, this is you making critical thinking and the evidence available in order to facilitate taking the proper decision…’*

##### 3.2.2.2. Subtheme 2.2: Some NMs Verbalised Feeling Overwhelmed by the Large Quantity of Evidence Available to Them and Having to Prioritise It

The NMs’ comments made it evident that there was ample access to an abundance of information from various information sources, including organisational and scientific information.

*‘… hospital intranet, online, everything on that site, you will see a lot of data, Salamtak (Electronic medical record) policies, procedures, the resources example, the E library, where you have the Lippincott Advisory…the dashboards that you require…’*

*‘…So at least four or five dashboards, we’ve got access to look at…we’ve just been bombarded with a lot of data and information every day. … how do we filter effectively, what can we prioritise to work on…you can’t see the wood for the trees. …what actually is the thing I should be looking at’*

*‘… I can say that the time we need time we don’t have enough time… There are lots of things researches we use everything …time constriction is a matter for us…’*

##### 3.2.2.3. Subtheme 2.3: NMs Require Training, Competencies and Skill in Using EIMgt

The NMs advocated strongly for training in EIMgt.

*‘…we don’t have scientific we don’t have statistics background so it’s not recommended from management and nurse manager or any management to be well educated and experienced how to read the data in a way what we need … this is for me a real challenge. Either they need me to educate me how to do it and if they educate me how to do it, I don’t have time to do it…’*

*‘…yes, we got the training, but the training itself, doesn’t didn’t seem well planned. And also the people presenting probably knew a little bit more than us, but not much more… so that’s the training side is the hindrance as well’*

*‘…I also did not use the library …’*

*‘… you need to be a tech, not everybody’s a tech. So that’s a challenge. You need to spend a lot of time initially to learn the dashboard, how it works, how you filter data, how you export data, what can go wrong, so that’s a challenge…’*

##### 3.2.2.4. Subtheme 2.4: NMs Shared Ideas on How to Develop a Framework to Use EIMgt

A striking observation is the diversification and individual originality in thought processes when demonstrating the expected characteristics of EIMgt.

*‘I brought the decision making in a circle in the middle. And as we go back to the definition of EiM, that it combines between managerial decision critical thinking and evidence… second one is patients’ needs … my experience…, values…’*

*‘… to ensure patient safety… I want to put it in the framework … We need to have a data and we need to have a resources so we can take evidence based information…’*

#### 3.2.3. Theme 3: EIMgt Improves OP

**Figure 4:**
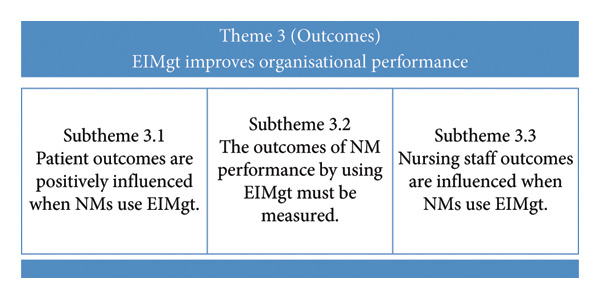
Summary of FGD Theme 3 and the subthemes.

The effect of utilising EIMgt on the organisation surfaced, whereby the overall OP is reflected in the outcomes on the patients, individual NM and nursing staff at the bedside. Figure 4 summarises FGD Theme 3 and the subthemes.

##### 3.2.3.1. Subtheme 3.1: Patient Outcomes are Positively Influenced When NMs Use EIMgt

The NMs discussed their participation and contribution to OP through achieving the target of these KPIs.

*‘…how are we doing with the care of Dialysis patients, pain audits, CAUTI [Catheter Associated Urinary Tract Infection] …’*

*‘… with the use of all this more data and information coming through is really improved efficiencies. So patient days are going up…bed occupancy is much more effective …length of stay has its moments… the information is there and it’s visible to everybody. People [staff] can be much better prepared to see how they’re doing with the nursing model of care programme, we have all that information visible on the units for the staff…’*

*‘…the rate of intraventricular brain haemorrhage was high…we looked at our practices and how to reduce it…evidence shows that if you put the babies in midline position for 72 hours…easy flow of blood…the brain is secured…prevents any brain bleeds…’*

##### 3.2.3.2. Subtheme 3.2: The Outcomes on NM Performance by Using EIMgt Must Be Measured

It is evident from the FGDs that NMs use information; however, what is unclear is how they use that information and its impact on their performance as NMs.

*‘…gives us a chance to compare ourselves with other hospitals organisations internationally… I know for example, with pressure ulcers, we are below the international benchmark for our organisation [lower is better] …’*

*‘…So when you benchmark with what can practice in your facility…this is …pushing you to, to do what they’re doing…to reach to with your resources …to deliver …pushing yourself to for the more success and you to meet your goals…’*

*‘…some people maybe they’ll be unsure how to get the resources who to go to for the resources before they’ll probably just give up rather than doing it…’*

##### 3.2.3.3. Subtheme 3.3: Nursing Staff Outcomes Are Influenced When NMs Use EIMgt

The premise established in this theme conveys the message that if the NMs practice EIMgt, staff outcomes are likely to be positively or adversely influenced. The lack of staff engagement, staff support and buy‐in was highlighted by NMs during the FGDs as potential barriers to using EIMgt due to a lack of a shared team approach to implementing changes or quality improvement.

*‘If you’ve got a team that are like not really supporting you, and they’re not really interested, … I’m going to share it [the information] with them and they’re not going to chase it…’*

*‘…engagement is not staff engagement… staff engagement in any projects [multidisciplinary projects] which may take a bit time…’*

*‘… there’s a sick leave dashboard…weekend sick leaves or public holiday sick leaves they [NMs] were monitoring…’*

## 4. Discussion

The study aimed to explore and describe NMs’ understanding and experiences using EIMgt in a healthcare facility in the UAE. Three main themes that emerged from the FGDs were related to structures, resources, and requirements for EIMgt. Additionally, the FGDs underscored the need for clearly defined processes or pathways required to guide EIMgt utilisation and highlighted the impact of these initiatives on OP outcomes. The three main themes were as follows: (1) NMs communicated resources and requirements to support using EIMgt practices; (2) NMs verbalised the need to establish a process or pathway to use EIMgt; and (3) EIMgt improves OP. Remaining true to the voices of the participants, their responses were described rather than being influenced by subjective interpretation.

### 4.1. Theme 1: NMs Communicated Resources and Requirements to Support Using EIMgt Practices

During the FGDs, NMs conveyed the much‐needed support in the form of hiring *(Position)* the correct people *(People)* as NMs with the relevant academic qualifications and experience *(Preparation)* and thus ensuring that NMs in their positions are empowered *(Power)* to perform their job functions. Additionally, having access to information sources and the availability of technology to collect information and make this information available to NMs in a meaningful way were key, according to the NMs. An extensive list of various sources of information with examples of types of information that NMs utilised routinely to carry out their functions was discussed. These include clinical performance indicators, patient satisfaction results and operational data metrics, such as length of stay, case‐mix index, nursing productivity and staff turnover. This information was readily accessible in a user‐friendly format, enhancing its ease of utilisation and establishing it as a go‐to source of information.

The NMs vigorously discussed contextualising information to their situation and setting before using it. They are alerted to the fact that evidence utilisation is not a ‘one‐size‐fits‐all’ approach and that a fair amount of customisation by the NM is required to render information relevant and meaningful for application [30].

NMs were empowered to function interdependently and demonstrated positive responses regarding their ability to implement initiatives to support changes in operational processes and organisational indicators. However, NMs were equally challenged and frustrated due to their inability to execute strategies or initiatives. At times, they were compelled to implement initiatives that did not align with their own perspectives. Escalating challenges and engaging in dialogue about these challenges with senior leadership were evident.

NMs are influenced positively or negatively by the engagement or lack of engagement of senior leadership and the bedside nursing staff. Senior leadership plays a crucial role in facilitating evidence‐informed practice by demonstrating commitment and support. They act as role models by championing and advocating for adopting the use of information [9]. Regular, open and transparent communication with middle managers paves the way for empowering NMs. Additionally, senior leadership’s commitment, engagement and advocacy are essential engaging behaviours that contribute to the use of information‐driven decisions.

### 4.2. Theme 2: NMs Verbalised the Need to Establish a Process or Pathway to Use EIMgt

It was evident that not all NMs were familiar with EIMgt, thus highlighting the need for clarity on its practical application in management practice. This emphasises the need for establishing a structured process or pathway to demonstrate this integration effectively. A combination of evidence and information, such as scientific and organisational data, information provided by customers and patients, managers and other professional expertise [9], is required by NMs as a consistent practice. NMs are aware of the limitless quantity of information available. However, the assimilation of multiple sources of information to obtain a well‐informed and well‐reasoned decision or strategy proved challenging for NMs. As a result, NMs often experience feeling overwhelmed, leading them to default to previous experiences or readily available information that is easily accessible [31]. NMs identified time limitations, the need to make quick decisions and conflicting priorities as barriers and information overload as contributing factors to the insufficient uptake of evidence. This shortfall could potentially have an adverse impact on the quality, time and efficiency of decision‐making [8, 10, 32].

The absence of clarity and knowledge on what EIMgt practice looks like is significant. Role modelling such behaviours for experienced and new NMs will be difficult. It is challenging to practice, and even role model behaviour is complex if NMs are unable to visualise it. The NMs suggested that having a framework with processes and a pathway that guides EIMgt practice will increase its uptake. Ideas from NMs on what a framework to improve the utilisation of EIMgt would look like emphasised characteristics, such as it being cyclical, continuous, scaffolded and relational to other concepts. These characteristics were consistent with the study on evidence‐based management in healthcare [33].

The NMs in the FGDs advocated strongly for training in EIMgt. Competence in utilising EIMgt encompasses the requirement for knowledge, skills and behaviours related to EIMgt. The skills required in EIMgt narrow in on the acts or processes of inquiry, seeking, appraising, analysing, applying and evaluating the various information available to NMs [30, 34, 35]. The NMs expressed the need for training in high‐level data analysis and understanding statistics.

### 4.3. Theme 3: EIMgt Improves OP

As middle managers, NMs are instrumental in facilitating the operations and functions of their clinical units. The necessity and value for NMs to use information to enhance the work environment of bedside staff, increase their individual capacity and capabilities as NMs, increase revenue and reduce patient infections. This practice strengthens their management practice and is expected to yield positive results in terms of improved patient outcomes, safety and quality of care. Ultimately, the overall performance of the organisation is positively influenced [1, 9]. There is a need for NMs to proactively use evidence and information to manage practice. Although a few NMs shared proactive implementation of information by adopting best practices, it was noted that utilisation of information was largely retrospective in response to problem‐solving or a need to improve clinical indicators not being met or manage change. In healthcare, remaining relevant and gaining and sustaining a competitive advantage are key challenges that healthcare leaders must overcome to assume market dominance. Therefore, it becomes a high‐priority goal for NMs to prioritise monitoring OP [36]. All organisations use information that may positively impact OP [37]. However, this is not the same as practising EIMgt, and there is a paucity of studies showing the correlation between EIMgt and OP and how leaders use this information within their context [1, 38].

Equally unclear is how NMs use information and its correlation and the impact on their performance as NMs, thus creating a need to measure EIMgt utilisation on NMs’ performance [30, 35]. In this study, there is no direct way to measure whether the NM is a more effective manager when using provided information. However, based on the NMs discussion, it appears that NM performance is indirectly related to the achievement of the organisations’ performance.

Drawing on best practices and evidence, NMs engaged bedside nursing staff in initiatives and unit‐based programmes such as quality link nurse programmes, the Comprehensive Unit‐based Safety Programme (CUSP) and the DAISY Award programme. They also engage in nurse well‐being programmes and participate in the NDNQI RN survey by Press Ganey. Efforts are made to cultivate a positive working environment supported by adequate staffing numbers and ratios, as well as reviewing and managing sick leave and unit safety trackers.

It was evident that NMs have a superficial understanding of EIMgt. Although they incorporate information in their management practices, they do not consistently and systematically seek, critically appraise, apply and evaluate evidence and information. The pertinent points of the main themes and related subthemes reinforced that for NMs to increase the quality and safety of patient care, which is ultimately their goal, the necessary structures and processes must be available and in place to enable EIMgt. Only then can NMs have a positive effect on self, patient, staff and organisational outcomes.

### 4.4. Limitations of the Study

Limitations of the study included the inherent bias associated with employing a qualitative research design. Furthermore, the study was conducted in a single site and with one group of healthcare professionals, necessitating further investigation at other sites and among other healthcare professionals to determine generalisability.

### 4.5. Implications for Nursing Management Practice and Education

This qualitative study offers valuable insights into the understanding and experiences of NMs in using EIMgt. Since there is a general lack of uptake of evidence to inform their management practice, the findings can inform recommendations to increase the utilisation of EIMgt, thereby fostering improvements in patient care quality and patient outcomes. The study recommends that effective EIMgt be continuous and incorporated into the daily management practices and routine of the NMs. It is important for EIMgt to be viewed and adopted as an integral skill, rather than a ‘go‐to’ tool for complex and challenging projects. NMs must role‐model the behaviours of EIMgt in practice so that RNs who may be prospective NMs may adopt such positive practices. A framework to enable and facilitate EIMgt by NMs should be developed to guide NMs.

### 4.6. Recommendations to Improve NMs’ Knowledge and Practice of EIMgt

Recommendations derived from the themes to improve NMs competency and practice in utilising EIMgt are classified into four main domains, namely: *Education* (skills in using evidence), *Infrastructure* (access to systems and information), *Culture* (organisation, leadership and incentives) and *Evaluation* (monitoring and measuring progress). Table 2 summarises these recommendations.

**Table 2:** Recommendations to improve NMs’ knowledge and practice of EIMgt.

Recommendations	Responsible personnel/level of implementation
*1. Education (skills in using evidence)*	
• Provide orientation and awareness of EIMgt and types of evidence and information available.	Hospitals’ education and training department—organisation level
• Provide quarterly workshops on critical appraisal of research evidence, organisational data, interpreting of benchmarking and performance reports.	
• Develop and introduce orientation and education modules on EIMgt, showcasing data‐driven and result‐oriented decision‐making in quality of patient care.	
• Role model and practice EIMgt skills in daily operations, utilise the Infinity LoopS_8_ Framework	NMs—individual level
• Develop a postbasic certification course on EIMgt to better prepare individuals for the position of NM	Hospitals’ education and training department in collaboration with affiliated University—organisation level and national Level

*2. Infrastructure (access to systems and information)*	
• Develop readily accessible information in easy‐to‐use formats for NMs such as digital dashboards, business intelligence platforms displaying real‐time organisational performance data and central repositories of best practices.	Hospital leadership, business, information and technology, quality and performance departments—organisation and system level.
• Integrate information into the organisations’ intranet for easy access.	
• Use a variety of forums such as staff councils, FGDs and patient advisory boards to gather information from diverse sources and utilise it to contextualise decision‐making and change management.	Hospitals’ senior (executive) leadership, nurse managers—hospital level

*3. Culture (organisation, leadership and incentives)*	
• Leaders to develop practice for NMs to submit ‘evidence to decision‐making’ when presenting proposals and change concepts, thereby embedding evidence utilisation into organisational culture.	Hospitals’ senior executive leadership, hospitals’ quality and performance department—hospital level
• Celebrate, recognise and reward units and NMs that implement and sustain evidence‐informed initiatives, thus strengthening the culture of EIMgt.	

*4. Evaluation (monitoring and measuring progress)*	
• Include an EIMgt index of performance measurement in the periodic and annual quality report and NM performance appraisal.	Hospitals’ senior executive leadership, hospitals’ quality and performance department—hospital level
• Report changes, projects and decisions made that impacted positively the quality of patient care and organisation performance.	NMs, hospitals’ senior executive leadership, hospitals’ quality and performance department—individual and hospital level

## 5. Conclusion

EIMgt is not a stand‐alone management practice or a reactive measure for addressing problems or making decisions. It rather constitutes a continuous, dynamic and iterative process that evolves and integrates itself within the daily management practices of NMs. EIMgt is touted for its potential to improve management practices and, in turn, enhances the quality of patient care, thereby directly impacting OP. Thus, resources are essential to support NMs in EIMgt practice. Establishing processes and pathways will enable EIMgt and ultimately contribute to improved OP.

## Conflicts of Interest

The authors declare no conflicts of interest.

## Funding

No funding was received for this manuscript.

## Data Availability

The data that support the findings of this study are available upon request from the corresponding author. The data are not publicly available due to privacy or ethical restrictions.
